# The impact of the design of payment scales on the willingness to pay for health gains

**DOI:** 10.1007/s10198-016-0825-y

**Published:** 2016-09-13

**Authors:** Lotte Soeteman, Job van Exel, Ana Bobinac

**Affiliations:** 1 Institute of Health Policy and Management, Erasmus University Rotterdam, iBMG, PO Box 1738, 3000 DR Rotterdam, The Netherlands; 2 Erasmus School of Economics, Erasmus University Rotterdam, Rotterdam, The Netherlands

**Keywords:** Payment scale, Willingness to pay, QALY, Uncertainty, Bias, Contingent valuation, Preferences, I10, I19

## Abstract

The questionnaire format applied in a CV study represents the way in which the WTP estimates are obtained. Payment scales are often used in CV studies as the questionnaire format of choice. The study summarized here analyzes the impact of the design of two payment scales (PS) on the monetary value of QALY gains. The scales differed in terms of their end-points, mid points, and coarseness. We judged the performance of the two PS against several indicators: the average WTP per QALY estimates, post-estimation uncertainty levels, the existence of mid-point concentration, and the dependency on end-points. Our results show that PS design influences respondents’ WTP values. The results also suggest that a more detailed scale with a more realistic range may help respondents to elicit values closer to their “true” WTP values, hence produce higher-quality outcomes. Further research and pretesting strategies are suggested to explore and minimize the effects of PS design on WTP estimates, which may ultimately increase the quality of WTP estimates.

## Introduction

Contingent valuation (CV) is a stated preference method that enables researchers to directly estimate the monetary value of a non-market good or service, either by asking respondents for their willingness to pay (WTP) for obtaining a good, or their willingness to accept (WTA) for giving it up (e.g., [15]). Many CV studies have been published in the field of health economics (e.g., [22]), of which a considerable number concerned valuing health gains (e.g., [7, 28, 34, 36, 42, 49, 53, 61, 68]). Although a carefully designed CV study can provide useful input for decision-making in healthcare (e.g., [14]), CV studies involve a number of methodological issues (e.g., [8, 25, 29, 46, 57, 63, 64, 66]). One of the design-related issues concerns the appropriate questionnaire format applied in CV studies. The questionnaire format refers to the technique with which the WTP estimates are elicited. The main questionnaire formats used in CV studies are the bidding game (BG), the dichotomous choice (DC) format, the open-ended (OE) format, and the payment scale (PS) format. Here we focus on the PS format, which was shown to have several advantages over the other questionnaire formats, as will be briefly discussed below.

Using a PS, an analyst presents a specified range of monetary values and asks respondents to select a value that best represents the amount they would be willing to pay for a specified benefit (e.g., [7, 56]). Mitchell and Carson [44] first proposed the PS and it has been used widely in different fields (e.g., [9, 20, 62]). Compared to BG and DC, PS avoids the starting point bias, since the question does not offer an initial bid to be used as an anchor, and avoids ‘yea-saying’, since a yes–no question is not posed [10, 16, 64]. PS can also conserve respondents effort because even a fairly detailed set of values offered on a PS can be visually scanned quite quickly, and given the simplicity of the question, there is no need for prompting by an interviewer [12]. PS can also reduce the high rate of item non-response in the OE format [21] since it is cognitively less demanding than formats not employing numerical cues. However, although they may reduce the cognitive burden, numerical cues offered on a PS provide respondents with a “comprehensible context” for eliciting their WTP values, which can significantly impact the outcome of a CV study [63, 66]. For instance, respondents may view the range of values offered on a PS as representing “reasonable” amounts for their WTP [45]. If, compared to the values presented on the PS, respondent’s true WTP value is relatively low, they may interpret it as being too low and, subsequently, choose to report a relatively higher WTP in a hypothetical exercise. The opposite of course is also possible. In this way, the PS range may result in respondents revising their true WTP estimates up- or downwards [66]. The more sensitive responses are to the provided ranges, the higher the likelihood of obtaining inaccurate WTP estimates [48].

However, to what extent are WTP estimates for health gains sensitive to the “comprehensible context” of PS design? Moreover, if WTP is sensitive to PS design, how can we discern which particular design of PS performs better? What can analysts do to reduce the dependency of WTP on PS design? The study summarized here was designed to explore these issues. Because there is no gold standard for designing the PS, improving our understanding of the effects different PS designs can have on WTP estimates, using different methods of pretesting PS scales, will reduce the uncertainty regarding the impact of PS on the final outcome of CV studies. Ultimately, since the majority of CV studies are undertaken to inform policy-making (e.g., [14]), more accurate WTP estimates can help improve decision-making based on social preferences.

Our exploration is in the domain of valuing health gains, expressed in terms of quality-adjusted life-years (QALYs). We start our exploration with an overview of the existing evidence on the effects of PS designs on WTP. We then formulate our hypotheses and explore the impact of two different PS designs on WTP per QALY estimates and analyze which design could be considered better, and why. Finally, we discuss our results and their implications for the process of pretesting payment scales in CV studies.

## Previous research and the contribution of the current study

Although many studies compared the performance of different payment formats on WTP estimates (e.g., PC vs. DC by [13, 32]; OE vs. PC by [21, 26, 30]), a relatively small number of studies directly explored the effect of different features of PS design on WTP estimates.^1^ In the area of environmental economics, two studies confirmed that PS end-points may influence WTP estimates. Rowe et al. [54] explored the differences in WTP estimates obtained using four otherwise-equal PS with end-points of $200, $1000, $5000, and $10,000, and found a significant difference in WTP obtained between the PS with the lowest end-point ($200) and those with the three higher end-points. Dubourg et al. [23] also reported a significant difference in WTP estimates between two PS with different end-points (£1500 and £500). The scale with a three times higher end-point yielded a 2.65 times higher average WTP value as compared to the scale with the lower end-point. The only study in the field of health economics that explored the effect of the design of PS on WTP estimates was the study by Smith [63]. This study focused on the impact of the ordering of PS value points on WTP estimates and showed that a PS with value points ordered from high-to-low increases the WTP as compared to a PS with either low-to-high or randomly sorted values.

Our study most resembles the study by Dubourg et al. [23], although there are important differences. First, Dubourg et al. [23] elicited WTP values from 94 respondents, whereas we use data from over 1000 respondents representative of the adult population of the Netherlands, which increases the reliability and generalizability of the results. Secondly, our study elicited WTP values in a two-step procedure, combining the PS with a follow-up OE question. Using a single PS, respondents were asked to first indicate the maximum amount they would definitely pay for a given QALY gain, then to indicate the minimum amount they would definitely not pay for this gain, and finally asked for their exact WTP in a bounded follow-up OE question. This OE-WTP was bounded by the minimum and maximum values indicated on the PS, i.e., by the “value gap” over which respondents were uncertain (e.g., [23]), and was taken as the estimate of individual WTP for calculation of WTP per QALY values.^2^ The two-step approach may be preferred to a single PS, for several reasons. First, the OE-WTP is elicited after considering the PS, arguably leading to more thought-through answers. Second, the approach generates a richer data set with multiple valuations per respondent. For our current study, the data allows us to test the impact of the design of PS both on the width and the position of the PS-WTP value gap, and on the final OE-WTP estimates (as described in the next section). Hence, the “goodness” of the PS scale design need not be inferred from the differences between the WTP point estimates obtained using different PS designs, but from the effect a particular PS design has on the respective OE-WTP or the value gap.

A final difference of this study with Dubourg et al. [23] was that along with every OE-WTP question we recorded the post-estimation response certainty surrounding the WTP estimates. Respondents were asked how certain they were about actually paying OE-WTP if asked right now, with response options: (1) totally certain I would pay; (2) pretty certain I would pay; (3) maybe yes, maybe no; (4) probably would not pay; (5) surely would not pay. The relationship between response uncertainty and WTP estimates is important because lab and field experiments have shown that WTP estimates accompanied by a higher level of response certainty better predict actual consumption behavior (e.g., [5, 6]) and the measure of uncertainty could be used to calibrate the hypothetical WTP and obtain the actual values (e.g., [2, 58]). It has also been suggested that issues such as range bias may be mitigated by restricting the analysis to the WTP values of those respondents who indicate they are ‘definitely sure’ they would pay their stated WTP [59], since these respondents may exhibit less anomalous behavior. Given the potential importance of response certainty, we explore whether a particular PS design fosters more response certainty.

Generally, comparisons between different payment scale designs may lead to two main outcomes. First, if we find no significant differences between PS, our focus may turn to understanding which scale is relatively most cost-effective to be used in surveys. If we find a significant difference between payment scales, then we must discern which scale design, if any, is preferred. By looking at comparisons beyond the mean WTP, this study attempts to address these questions.

## PS design and hypotheses

**Fig. 1:**
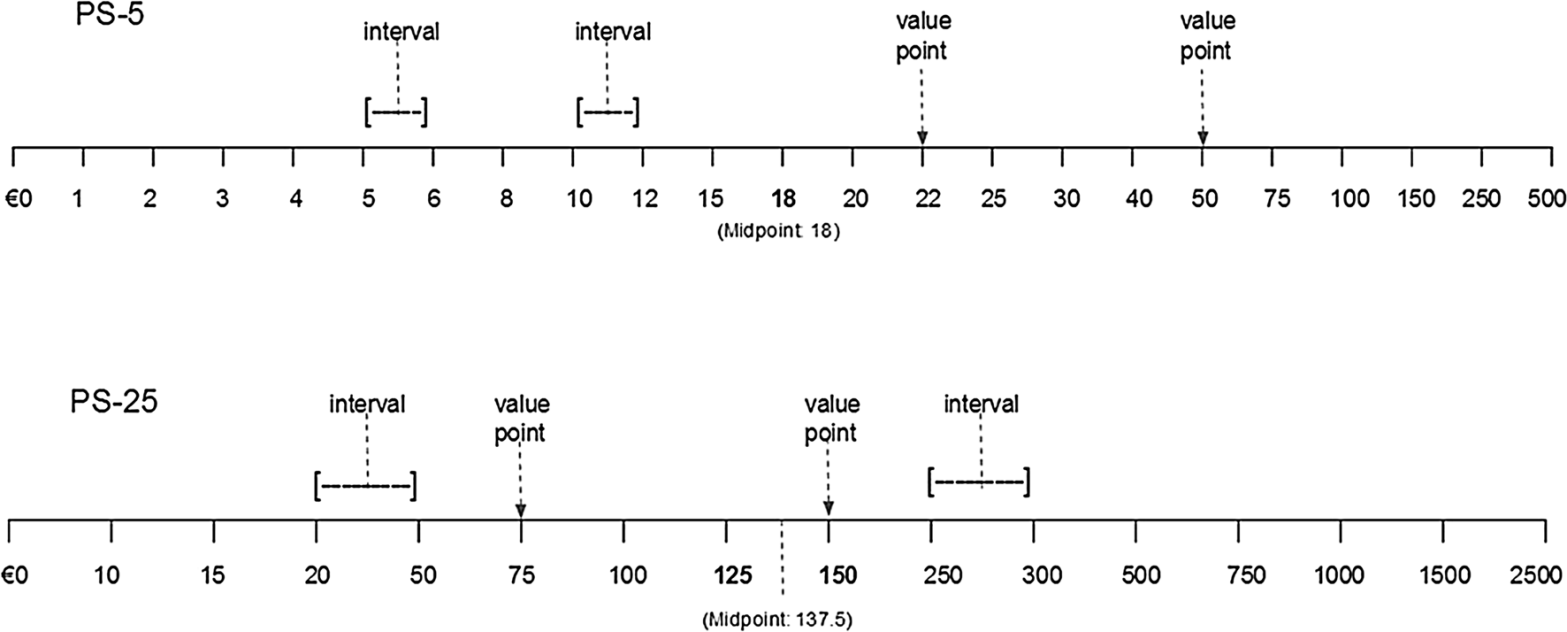
PS-5 and PS-25: intervals and value points

We designed two payment scales, labeled PS-5 and PS-25 (Fig. 1) and randomly assigned 1015 respondents to either PS (details of the design and sampling are presented below). The payment scales accompanied otherwise identical WTP questions and a two-step approach was applied to elicit WTP, as described above, yielding the following estimates of the QALY gain on offer (Fig. 1):

- PS-5_L,A_ and PS-25_L,A_ indicating the average maximum amount a respondent would pay (lower bound of the value gap);
- PS-5_U,A_ and PS-25_U,A_ indicating the average minimum amount a respondent would not pay (upper bound of the value gap);
- OE-5_A_ and OE-25_A_ indicating average OE-WTP.

PS-5 and PS-25 mainly differed in terms of their end-points (€500 in PS-5 vs. €2500 in PS-25) and the number of value points (23 points on PS-5 and 16 on PS-25), making PS-5 a considerably more detailed (less coarse) scale (Fig. 1). PS-25 covered a wider range with fewer value points, i.e., the average size of the interval between two value points was larger for PS-25 than PS-5. The intervals between value points were unevenly distributed along both scales, with considerably wider intervals towards the end of the scales. As a result, the mid-points of each scale differed from the mid-point values (Fig. 1). Finally, PS-5 offered a value point located exactly at the middle of the scale (i.e., the 12th value point) whereas the middle of PS-25 was located between two value points (i.e., the 8th and the 9th point).

Based on the points at which WTP values were elicited, we formulate our hypotheses:

- H1: Differences in design between PS-5 and PS-25 lead to statistically different average WTP and WTP per QALY estimates, with PS-25 leading to relatively higher OE-WTP values.

There is evidence that the average WTP values are correlated with the PS end-points (e.g., [23, 54]). We first test whether the difference in WTP estimates are related to the difference in the end-points of the scales.

- H2: Differences in design between PS-5 and PS-25 lead to a difference in response patterns.

Beyond testing the equivalence of mean WTP amounts, this study looks at additional comparisons, such as variance, frequencies, distributions and the presence of extreme values, along with response rates, item non-response and proportion of protest responses—all additional issues to consider when comparing the performance of two payment scales.

- H3: Differences in design between PS-5 and PS-25 lead to difference in the central tendency of WTP values and therewith to mid-point centering of OE-5_A_ and OE-25_A_ estimates.

If respondents have stable, well-formed preferences, their WTP value is expected to be independent from the PS design. However, in case preferences are not stable or well formed, respondents may resort to different heuristics and construct a WTP value on the spot. They may use the mid-point of the scale (€18 in PS-5 and €137.5 in PS-25, Fig. 1) as a cue for forming a value. We investigate whether and how respondents use the mid-point of the scale to form their WTP values, and analyze the central tendency of WTP responses.

- H4: Differences in design between PS-5 and PS-25 are associated with different levels of self-reported response uncertainty, captured by the width of the respective value gaps and the post-estimation certainty levels.

Arguably, a particular design of the payment scale may be considered a better or worse vehicle for expressing preferences. All other things being equal, one PS may foster more certainty in stated preferences, for instance because it offers numerical cues that respondents can “work with” (for instance, familiar values) and hence be of help in expressing, or constructing, their preferences. Keeping other important determinants of post-estimation response uncertainty constant (sample representativeness, wording of questions, payment vehicles, etc.), we investigate the association between the self-reported uncertainty (i.e., size of the value gap [31] and the distribution of response uncertainty) and PS design.

## Methods

A sample of 1015 respondents representative of the Dutch population according to age (18–65 years), gender, and education participated in this study (Table 1). The data was collected through an online questionnaire as a part of a wider study exploring the value of a QALY. The questionnaire (see Appendix 1) asked respondents to solve six WTP questions in total, including the question analyzed here, which was presented as the third question. Respondents were asked to value an individual (own) QALY gain described by a difference between two EQ-5D health states [24]. In total, 42 health states were combined into 29 scenarios, which were presented to respondents at random, respecting scenario balance (Appendix 3).^3^ Respondents were first asked to indicate which of the two health states they considered as better and then asked to imagine being in the better health state, but facing the risk of spending 1 year in the worse health state (i.e., either a 2, 4, 10, or 50 % risk), starting the next day. The concept of risk was graphically explained to respondents at the beginning of the survey (Appendix 2).^4^ The risk of the personal health decrement could be reduced to zero by taking a painless medicine once a month during the period of 1 year. The medicine would have to be paid through an increase in their health insurance premium, also during the period of 1 year (i.e., in 12 monthly installments). Respondents were reminded to take their household budget into consideration as well as which elements of the budget (e.g., rent, food, clothing, entertainment, education) they would need to economize on. Moreover, in the introduction of the questionnaire, respondents were told that healthcare decision-makers want to spend the healthcare budget in the best way possible, and in order to do that, they are interested in how people value different health states. It has been suggested in the literature (e.g., [11, 40, 41]) that “consequentialism” and “cheap talk” approaches may reduce hypothetical bias. For further details of the study design, we refer the reader to the published results in Bobinac et al. [8, 9].

**Table 1:** Summary statistics

Variable	PS-5				PS-25			
	Mean	SD	Min	Max	Mean	SD	Min	Max
Age (years)	42.2	13.1	18	65	39.3	12.4	18	65
Sex (female)	0.49	0.50			0.51	0.50		
Married (yes)	0.62	0.49			0.55	0.50		
Children (yes)	0.55	0.50			0.46	0.50		
Number of children^a^	2.07	0.93	1	5	2.06	1.26	1	15
Higher vocational or academic education (yes)	0.34	0.47			0.36	0.48		
Employed (yes)	0.60	0.49			0.64	0.48		
Household income (€)	2724	1694	999	10,000	2563	1501	999	10,000
(% < €1000)	0.12				0.15			
(% ≥ €1000 and < €2000)	0.34				0.34			
(% ≥ €2000 and < €3500)	0.37				0.37			
(% ≥ €3500)	0.16				0.14			
Number of people living on household income	4.25	2.72	1	13	4.01	2.53	1	13
EQ-5D (Dutch tariff)	0.85	0.23	0	1	0.85	0.23	0	1
EQ-VAS (1–100)	72.7	19.6	0	100	72.5	18.3	0	100

*VAS* visual analogue scale

^a^PS-5, *n* = 583; PS-25, *n* = 485

The expected QALY gain was calculated as the difference between the utility weights of health states 1 and 2 presented to respondents in each scenario, multiplied by the level of risk. In the 29 scenarios, subjects valued expected QALY gains ranging from 0.002 to 0.066; the average size of the expected QALY gain did not differ between the versions offering PS-5 and PS-25 (*p* > 0.05, Table 2).

**Table 2:** QALY gain, PS value range, and OE-WTP (monthly; in €)

Variable	PS-5		PS-25		*p*
	Average	SD	Average	SD	
PS_L,A_	36.98	79.18	115.77	312.75	0.000
OE-WTP	53.36	91.91	154.21	351.27	0.000
PS_U,A_	115.28	167.78	358.56	675.60	0.000
Expected QALY gain	0.087	0.148	0.096	0.165	0.054
WTP per QALY (per year*)	227,200		404,400		0.000
*n*	508		507		

* Monthly values multiplied by 12

WTP per QALY estimates were calculated as a ratio between OE-WTP and the expected QALY gain, for each row of the data. Hence, all PS and OE-WTP values qualify as “raw” WTP values, as they were read directly from the questionnaire, unlike the WTP per QALY values, which are a product of calculations.

Wilcoxon test (two independent samples) and paired *t* tests were used to determine statistical difference between the relevant values. To compare PS performance in terms of response, *z*-tests were conducted to test for equal proportions between the two payment scales. To explore whether the range of the PS had a direct effect on WTP estimates, ceteris paribus, a multivariate regression on PS-5 and PS-25 pooled data was performed, with OE-WTP as the dependent variable:

$${\text{OE}} {\text{-}} {\text{WTP}}\, = {\kern 1pt} \,\beta_{0} \, + \,\beta_{1} \left( {\text{expected QALY gain}} \right)\, + \,\beta_{2} \left( {\text{age}} \right)\, + \,\beta_{3} \left( {\text{income}} \right)\, + \,\beta_{4} \left( {\text{education}} \right)\, + \,\beta_{5} \left( {\text{gender}} \right)\, + \,\beta_{6} \left( {\text{dPS}} \right)\, + \,\varepsilon .$$

While controlling for the expected QALY gain and respondents’ income, the significance of dPS variable would confirm the direct effect of the design of PS on respondents’ maximum OE-WTP. All variables were tested for the normality of distribution using Shapiro–Wilk test and graphic interpretation of the Q–Q plot; if variables were not normally distributed, these were log-transformed. Multicollinearity between variables was analyzed using Pearson’s correlation coefficients. The data was analyzed using STATA 11.

## Results

The yearly OE-5_A_ was €636 (12 × 53) and OE-25_A_ was €1848 (12 × 154) (*p* = 0.00, Table 2), about three times higher. The WTP per QALY was €277,200 from PS-5 and €404,400 from PS-25 (*p* = 0.00, Table 2), which is approx. 55 % higher,^5^ confirming H1. Similar results were obtained from the regression analysis (Table 3, model 1).^6^ When controlling for other important determinants, PS-25 yielded a 245 % (i.e., exp^0.896^) higher OE-WTP value than PS-5 (*p* = 0.00), along the lines of the uncorrected results reported in Table 2. The conclusions of model 1 do not change when risk and health gain are separately included in the regression (although it shows that risk level 50 % had the highest positive influence on OE-WTP) (model 2).

**Table 3:** Results of multivariate regression analysis with Log(OE-WTP) as the dependant variable (*n* = 936)

Variable	Model 1			Model 2			Model 3		
	Coefficient	SE	*p*	Coefficient	SE	*p*	Coefficient	SE	*p*
log(expected health gain^a^)	0.128	0.027	0.000	0.861	0.028	0.003	0.09	0.023	0.000
Age	−0.021	0.004	0.000	−0.199	0.003	0.000	−0.019	0.003	0.000
log(income)	0.697	0.098	0.000	0.578	0.088	0.000	0.531	0.089	0.000
Education (high = 1)	0.146	0.108	0.177	0.024	0.031	0.41	0.032	0.031	0.295
Gender (female = 1)	0.180	0.101	0.075	0.141	0.089	0.115	0.132	0.089	0.141
Payment scale (PS-25 = 1)	0.896	0.100	0.000	0.917	0.089	0.000	0.842	0.165	0.000
Constant	−1.311	0.769	0.089	−0.735	0.680	0.281	−0.048	0.687	0.944
Risk 2 %				Omitted					
Risk 4 %				0.095	0.126	0.448			
Risk 10 %				0.109	0.132	0.408			
Risk 50 %				0.305	0.124	0.015			
PS*certainty level							0.077	0.167	0.644
*R* ^2^	0.172			0.191					

^a^The level of health risk presented in scenarios is a part of the expected QALY gain, which is a multiplication of the level of risk and the size of the health gain (or the difference between the utility weightings of the two EQ 5D health states offered in each scenario)

**Fig. 2:**
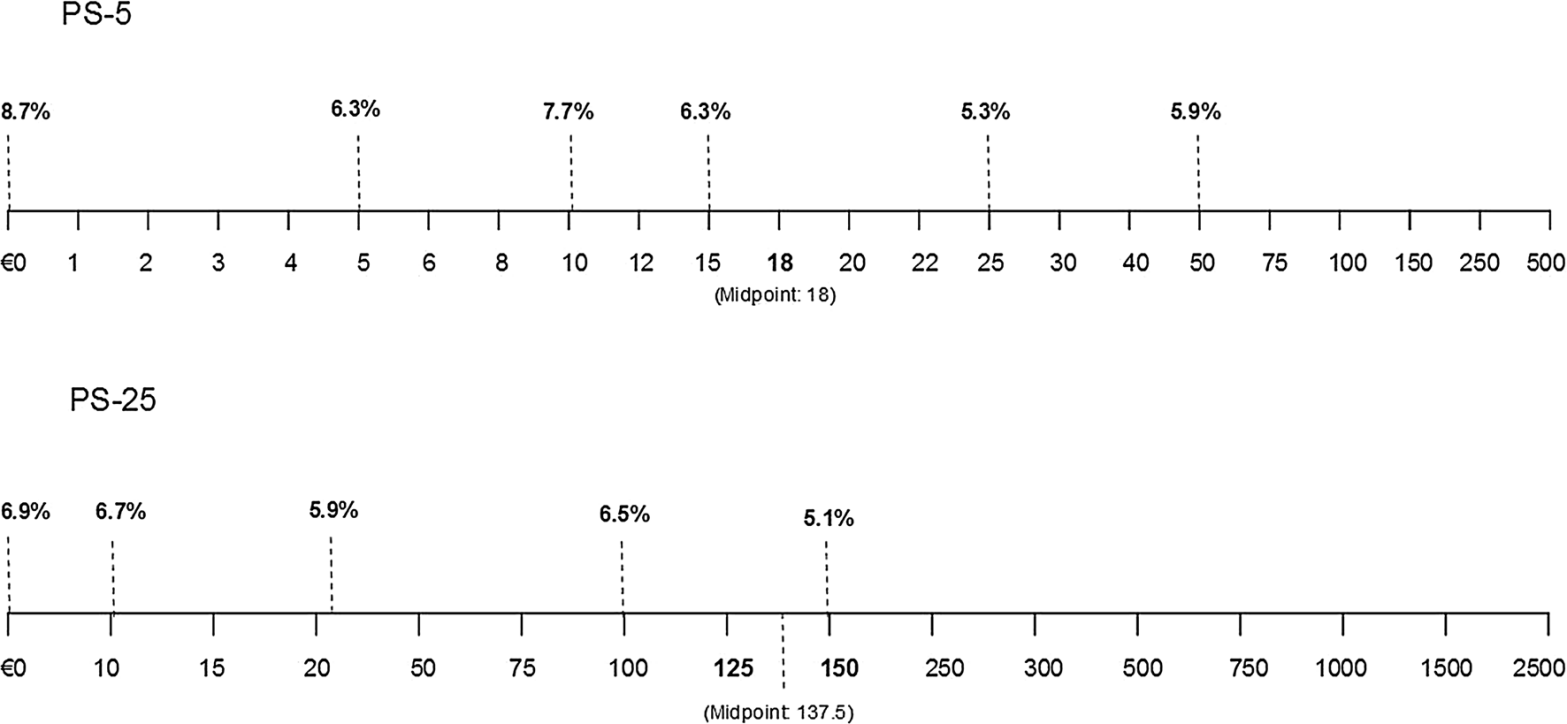
Most frequently stated maximum WTP OE, obtained following PS-5 and PS-25 (here presented on PS-5 and PS-25

In terms of response patterns (H2), there is mixed evidence. On the one hand, there was no significant difference between the number of zero responses or response time between PS-5 and PS-25 (*p* > 0.05), and less than 2 % of respondents indicated zero WTP using either scales (zero responses were retained in the analysis). On the other hand, the distribution around the means in PS-5 was smaller than in PS-25 (Levene’s test for the homogeneity of variances significant, *p* < 0.05), and less than 4 % of respondents opted for an OE-25_A_ in the upper quarter of the value range of PS-25 as compared to 27 % of respondents solving PS-5 (*p* < 0.05). The number of prototypical, rounded values (ending in 5 or 10) stated in OE-25_A_ was considerably higher than in OE-5_A_ (37 > 13 %, *p* < 0.05) (Table 4). Non-rounded values are, on the other hand, quite similar in terms of frequencies between PS-5 and PS-25. 78 % of OE-5_A_ values were equal to value points offered on PS-5, relative to 51 % in PS-25 (Table 4; Fig. 2). Finally, larger intervals between the value points seemingly lead to rounding. In PS-5, after the amount of €250, all respondents rounded their WTP to the nearest multiple of €50 (i.e., €350, €450). In PS-25, after the amount of €750, all respondents rounded their WTP to the nearest €100 (i.e., €1400 or €1600).

**Table 4:** Frequencies table (PS-5 and PS-25)

	PS-5		PS-25	
	Freq.	%	Freq.	%
Amount (already) on scale (value point)	395	77.8	258	50.9
Non-rounded amount (not on scale)	47	9.3	60	11.8
Rounded amount (not on scale)	66	13.0	189	37.3
*n*	508	100	507	100

**Fig. 3:**
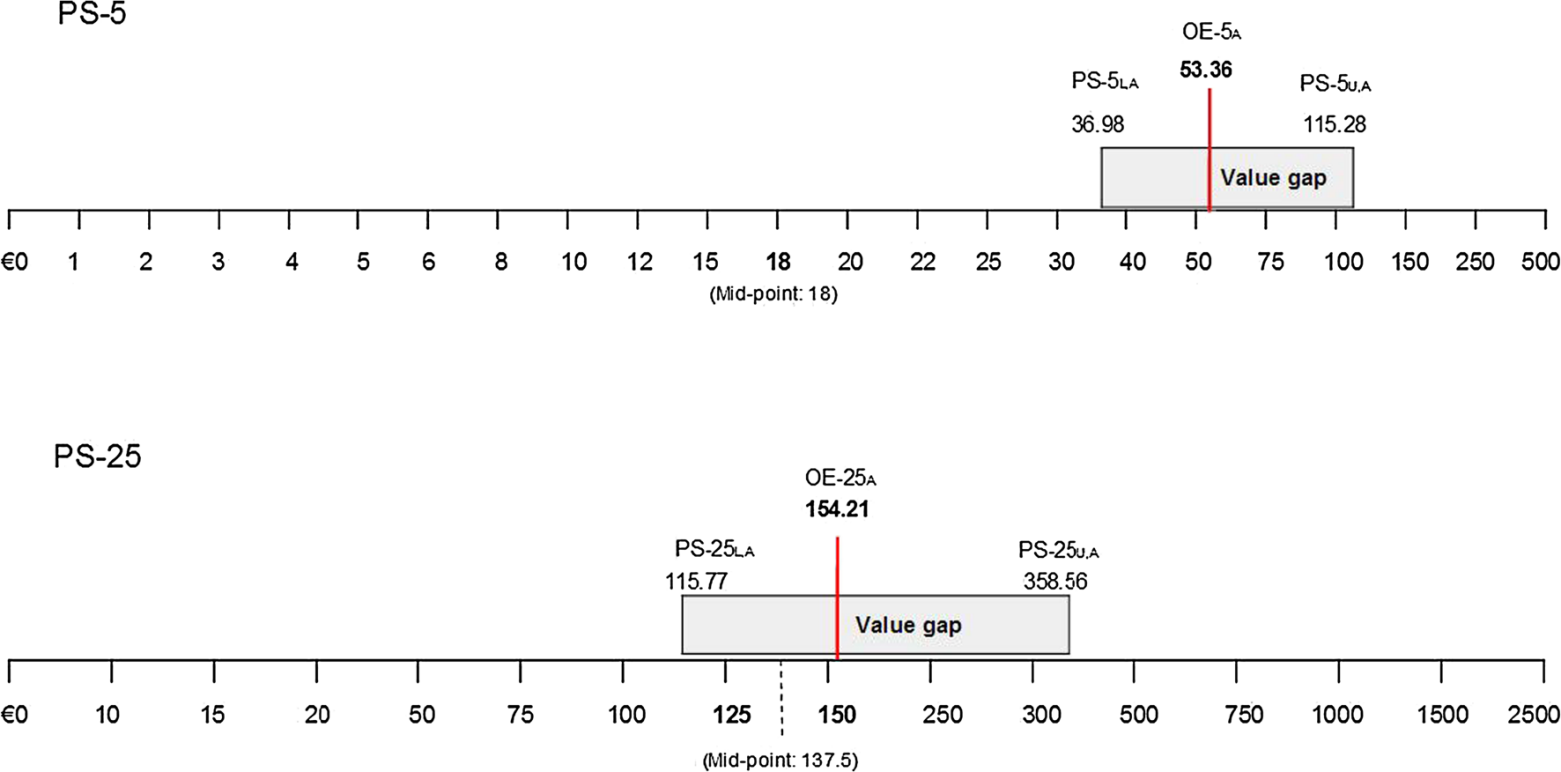
Respondents’ WTP value gaps and related maximum OE-WTP (monthly values*) Note: *L* lower end of the value gap; *U* upper end of the value gap; *A* average. *To obtain yearly values, monthly values should be multiplied by 12

Figure 3 presents the average value gaps and the corresponding OE-25_A_ and OE-5_A_. PS-25 yielded values that were clearly concentrated around the mid-point; OE-25_A_ fell almost at the centre of the PS-25_L, A_–PS-25_U, A_ value range. When deleting extreme values at (or beyond) the very extreme of PS-25 (>€2000; *n* = 7), the monthly OE-25_A_ was €122, which is almost exactly in the middle of PS-25. On the contrary, PS-5 did not result in a high concentration of average values around the mid-point, hence confirming H3.

**Fig. 4:**
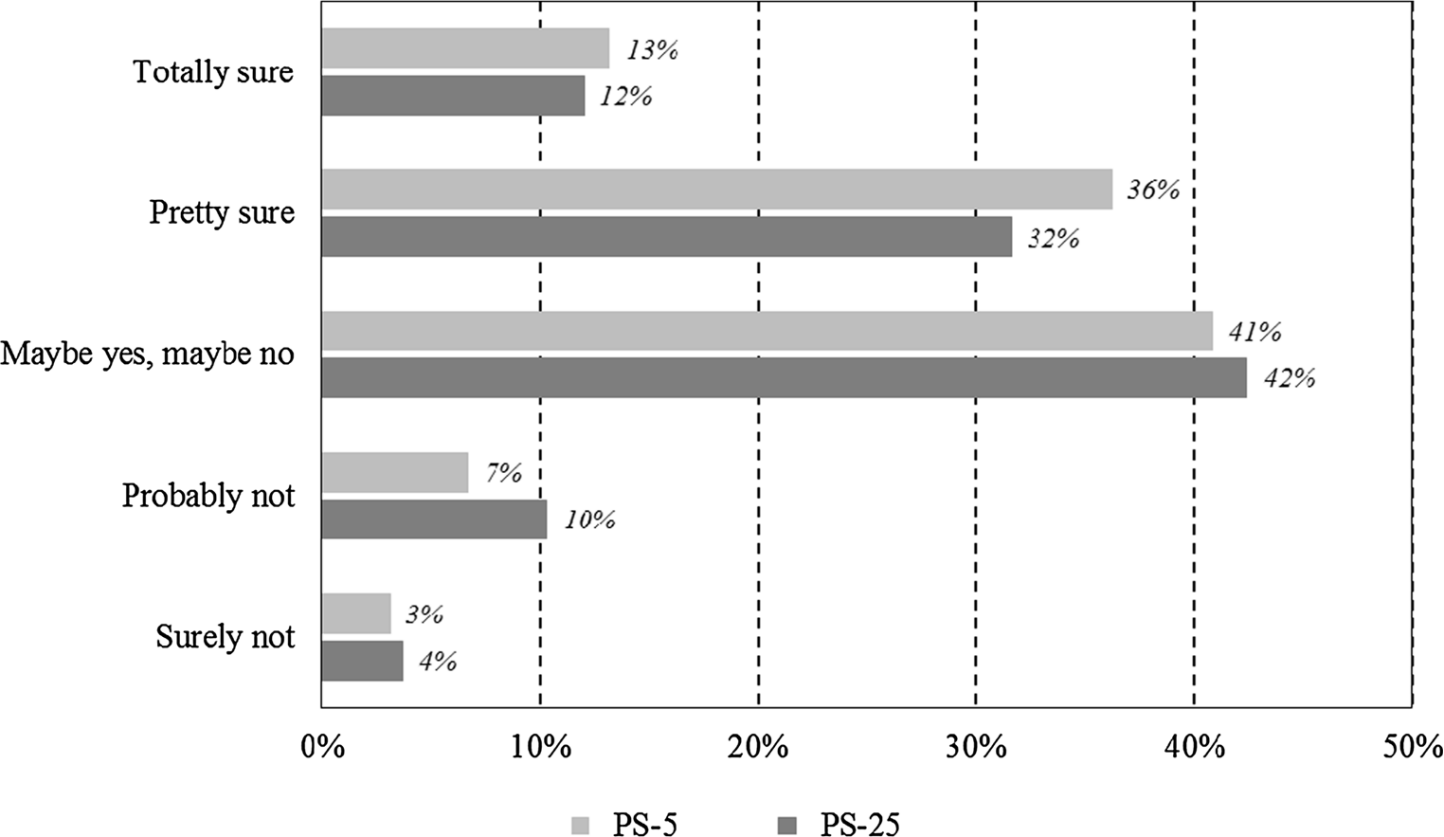
Response certainty

Respondents who used PS-5 reported relatively more certainty regarding their hypothetical WTP values, both in terms of narrower value gaps and in terms of post-estimation uncertainty, revealing some support for H4. The average value gap for PS-5 was €78 and for PS-25 was €243 (*p* = 0.00, Fig. 3). This is approx. a factor three difference, similar to the factor three difference in OE-WTP. If the width of the value gap is taken as an indication of preference uncertainty (where wider gap = more uncertainty), then the level of response certainty was on average higher in PS-5 than PS-25. Similarly, the post-estimation self-assessed certainty was also higher following PS-5 than PS-25 (*p* = 0.038). A somewhat higher proportion of respondents were pretty sure or totally sure that they would pay the OE-5 if they had to do so right now, relative to OE-25 (Fig. 4). The correlation between the width of the value gap and the self-reported certainty is, however, negligible (*r* = 0.1, *p* = 0.018 in PS-25 and *r* = 0.01, *p* = 0.7 in PS-25), indicating that the two methods of capturing uncertainty may not be representing the same underlying preferences.

There is no correlation between OE-WTP and post-estimation certainty (*r* = −0.018, *p* = 0.55). However, to test whether the conclusion regarding the relationship between PS design and OE-WTP changes when we explicitly account for preference uncertainty, we included an interaction between the PS dummy and the level of post-estimation certainty in model 3 (Table 3). Although post-estimation uncertainty assessment was performed after OE-WTP was elicited, it may reflect some level of inherent respondents’ uncertainty, which may drive the OE-WTP instead of (or alongside) the PS design. However, the interaction term is insignificant, indicating that the association between the OE-WTP and the PS design is likely independent from response uncertainty when uncertainty is measure using post-estimation self-assessment.

## Discussion

This study explored the sensitivity of WTP estimates for health gains to PS design. If respondents have stable, well-formed preferences, the WTP value they express is expected to be independent from the PS design, or any other aspect of a contingent valuation question. However, in case preferences are not stable or well formed, the resulting value may be influenced by the characteristics of the PS. The findings described in this paper confirm that the outcome of a contingent valuation study employing a payment scale can indeed be influenced by the design of this payment scale, to a considerable extent. Although we have illustrated this sensitivity with just one WTP question, it is likely that the choices regarding the payment scale (i.e., range, intervals, distributions of values, etc.) will be a fundamental issue in any contingent valuation study.

Non-market valuations are necessary in certain circumstances, for instance when revealed preferences are not available. Different WTP per QALY estimates used in a cost-benefit analysis may lead to different conclusions of the social welfare impact of an intervention. Hence, it would be constructive to think of how survey methods can be improved in order to obtain results that are more reliable. In the context of this study, it would therefore be relevant to discuss whether PS-5 or PS-25 performed “better”, and if so, why. To make inferences about the “goodness” of PS-5 and PS-25, we tested different features of WTP estimates obtained using the two scales.

In particular, PS-5 yielded OE-5_A_ values surrounded by relatively more response certainty, suggesting that it may have increased reliability relative to PS-25. We base this argument on previous experimental evidence suggesting that WTP values surrounded by more certainty correlate better with the actual, or observed, consumption behavior (e.g., [6]). If the degree of accuracy of WTP estimates can be measured by the strength of correlation between the WTP and actual consumption behavior, higher levels of post-estimation certainty and narrower value gaps may provide an indication of the “goodness” of the PS. Here, “better” means more accurate, and according to the certainty criterion, PS-5 appears to be the better scale.

Moreover, the larger central tendency of the PS-25 and OE-25 estimates may also be taken as indication the PS-25 is a poorer vehicle for preference expression. Generally speaking, a neutral mid-point in the response scale can serve as an anchor point to respondents [3, 47], especially for respondents whose preferences are not well formed [52]. Given the similarity of our split samples and the health gain sizes on offer, one interpretation of the central tendency of PS-25 is that this PS led more respondents who were uncertain about their WTP to base their valuation on the “easy cue”—the mid-point.

The question now is why PS-5 would perform better in this setting. First, smaller value points presented on PS-5 may better reflect the context of payments through health insurance, described in our contingent market. Respondents may be more familiar with smaller values in their daily reasoning, for instance when thinking of health insurance premiums, and hence be better at discriminating between values in the lower end of the value range, which were better represented in PS-5. Second, PS-5 is a less coarse scale, i.e., a scale with a higher number of value points and (relatively) smaller intervals, and more exact values. Coarseness is important because the respondent uses the PS to convert (or map) her true WTP into a position on the PS, and if the scale is too coarse it may lead to information loss and provide a less accurate reflection of ‘true’ values^7^ ([1, 51, 55]). In other words, if PS is characterized by a higher degree of exactness, it may evoke more exact OE-WTP values (e.g., [67]). However, although PS-5 was a less coarse and a more exact scale, and hence perhaps a better vehicle for expressing respondents’ preferences, the question of how many scale points is optimal remains unsolved [60]. Analyzing the scales used to report respondents’ attitudes (not WTP), Goggin and Stoker [27] found that the costs of employing an unduly coarse measure are significant, in terms of lowered reliability, validity, the associated biases and power limitations in statistical estimation. Although measures that are needlessly coarse and those that are needlessly fine-grained each have their problems, “scholars should err in the direction of seeking more fine-grained rather than less fine-grained measures” [27].

Although optimal PS design for every specific CV context may remain unattainable, we may considerably improve our designs and survey instruments by careful scale pretesting––and the same could be said for all other questionnaire formats. Pretesting is crucial because, as this study shows, the resulting value of a health gain can be manipulated by decisions regarding the payment scale, which decreases the usefulness of CV research. So far, however, the pretesting of payment scales seems largely confined to exploring whether unrealistically high end-points were present on the scale (which is judged by observing frequencies with which highest values are chosen, e.g., [8]). We argue that additional criteria should be introduced, such as sensitivity to mid-points or the width of value gaps. Pretesting could identify the approximate marginal distribution of values in the population and could avert the use of inappropriate payment scales. Several PS may need to be pretested while designing a CV study (and not a single scale, which is then collapsed or extended, depending on frequency testing; e.g., [9]), and a description of pretesting procedures should be provided for evaluators of contingent valuation studies. Once the analyst observes that the scales are largely insensitive to, for instance, the end-points and mid-points of the scale, and that post-estimation certainty increases and value gaps narrow, she could be more confident in using the scale. Work from other areas may also be very helpful in designing better PS scales (e.g., [65]) and devising protocols for scale pretesting. Pretesting should reduce the dependency of WTP on PS design and increase the reliability of WTP estimates.

One of the main limitations of our study is our inability to fully distinguish between the effects of each feature of PS on WTP, due to the multiple differences between PS-5 and PS-25. We cannot exclude a possibility of a combined effect of different scale features, nor can we be certain which feature of PS is most prominent. The aim of this study was not to single out the effect of each particular feature of PS on WTP, but to show how two distinct designs can lead to considerable differences in WTP. It would be interesting to repeat this research in a different setting where each of the features of PS could be investigated independently, preferably in an experimental study involving actual payments. It would be interesting to test the impact of PS design on WTP values for more familiar, “every-day” goods. Placing a monetary value on a health gain is a difficult exercise. Although the study design strived to help respondents understand the gain under valuation (e.g., using graphical explanations), it is still possible that the PS design would have had less effect on the monetary value of a familiar good, due to known reference prices, better-formed preferences or experience in trading. For instance, a WTP exercise aimed at valuing a new type of bread may not be as affected by the PS design as the value of a QALY was, which may reduce the generalizability of our findings. On the other hand, the WTP question analyzed in this paper was the third consecutive question presented in the online contingent valuation study, following two very similar WTP per QALY questions (reported in [9]). The difference between the third and the preceding two questions was only in the payment method (out-of-pocket vs. health insurance) and the experience respondents gained by solving two initial WTP questions may have somewhat reduced the unfamiliarity with the good under valuation and the valuation process itself, which may have a positive impact on the generalizability of our findings. This is, however, difficult to test. Understanding exactly how respondents perceive and complete payment scales could further help the development of PS yielding more reliable WTP estimates.

A second limitation of this study may lie with the data collection method. We used an online survey, which limited our ability to foster respondent engagement or reflection while solving the questionnaire. If preferences are constructed or learned during survey completion (especially for unfamiliar goods, e.g., [4, 43]), online surveys may provide highly contingent results [50] that may not be close representations of the “true” underlying values. In terms of engagement and reflection, face-to-face interviews have been the recommended as the “gold standard” [45]. However, recent research exploring the effects of different survey modes on how preferences are formed and stated (e.g., [18, 19]) shows in fact that the data obtained from online surveys and face-to-face interviews are not substantially different (further discussion in [39]). On the other hand, the online survey mode has advantages, such as relatively easy access to geographically spread respondents at lower cost, the opportunity to use interactive designs and graphical illustrations and so create more easily understandable studies, as well as allowing respondents to answer in their own time. Still, issues like population representation in an online panel should be further addressed since this is important for delivering reliable welfare estimates for social policy assessment. Thirdly, examining predictive validity or test–retest reliability may lead to different conclusions about the efficacy of the PS than what we present here, and experimental evidence may add to the reliability of our findings. Finally, while this study cannot fully discern all the mechanisms leading to different WTP per QALY estimates obtained using different payment scales, and some mechanisms may be working in combination or in different directions, it does reveal that the design of payment scales is not a choice to be taken lightly. It is hoped that this article will stimulate researchers to improve PS design. Each research context may even require its own, a priori unknown, “optimal scale” and it is therefore important to test the appropriateness of several PS designs before learning what is the optimal type of scale for a particular context. Pretesting procedures are thus important to reach correct interpretations and valid inferences and hence improve welfare assessment based on social preferences measured using contingent valuation. For policy-makers the results of this study are important because they show how manipulations can affect the results of a CV study, and therefore how important it is to understand the determinants of (the reliability of) WTP values.

## Appendix 1: Example of a WTP question from the questionnaire (translated text and screen prints)

[Introduction to WTP questions] “Imagine that you are currently in the health state you just described as better and that tomorrow you face going to the health state you described as worse for a period of 1 year. After this year experiencing the worse health state, you will return back to the better health state. Now, rather than spending a year in the worse health state, you could avoid this and remain in the better health state instead. For this, you will have to take a painless pill each month during that year. You will have to pay for these pills yourself, from your (household) income, through an increase in your health insurance premium. Have your ability to pay (given your household income) in mind!!”

The text in the text boxes provides a description of the two health states, using EQ5D-3L classification, which varied between scenarios. In this example, the translation of the health states is: (for the top text box) I have no problems walking about, I have no problems dressing or washing myself, I have no problems with daily activities, I feel mild pain or discomfort, I am mildly depressed or anxious; (for the bottom box) I have some problems walking about; I have some problems dressing or washing myself; I have no problems with daily activities; I feel mild pain or discomfort; I am mildly depressed or anxious.

Payment scale format, lower bound. “Suppose you would have to pay an amount for this pill right now. Please consider the range of amounts below. Now, start from the left and tick the highest amount you would definitely pay for this pill on a monthly basis for the duration of 1 year to avoid going to the worse health state.”

Payment scale format, upper bound. “Next, continue moving up the line and tick the first amount you would definitely not pay for this pill on a monthly basis for the duration of 1 year to avoid going to the worse health state.”

Open-ended format. “You have indicated that you would definitely pay €50 and definitely not pay €150 to avoid experiencing the worse health state for 1 year and remaining in the better health state. Please write in the amount (between €50 and €150) that most closely approximates the maximum you would be willing to pay per month to avoid going to the worse health state?”

Certainty level: “You have indicated that you would definitely pay €50 and definitely not pay €150 to avoid experiencing the worse health state for 1 year and remaining in the better health state. How certain are you that you would pay the stated amount, if asked to do so right now?”

## Appendix 2: The graphical explanation of the concept of risk

A 10 × 10 matrix of green dots represented a hundred people—each dot being an individual “just like you”. To demonstrate the meaning of, say, a 40 % chance of becoming ill, we asked respondents to click on one of the green dots (clicking superimposed a black “x” on the dot). Respondents were told that the computer then randomly selected 40 of the hundred dots and turned them red; the chance that the dot they marked would turn red was 40 in 100, or 40 %. The same example was repeated with a 1 % chance.

Translation from Dutch: “Now imagine that in this group of 100 people the probability that someone becomes ill rises to 40 %. In other words, 40 people will become ill and 60 people will not. To get an idea of what it means for the probability that you will be one of the people becoming ill, choose a green dot and click on it. The probability of the dot you selected changing color is 40 to 100.”

## Appendix 3: Design of the choice scenarios, levels of risk, and expected QALY gain

**Table:**

Choice scenario	Health state 1	Health state 2	Level of risk (%)
1	22222	11131	10
2	33232	33323	50
3	21312	12111	2
4	22323	21312	2
5	22323	12111	2
6	21232	32211	4
7	11112	22121	10
8	11122	22122	10
9	21323	22233	4
10	22331	21133	4
11	21111	12121	50
12	23232	32232	50
13	11312	11113	10
14	12311	11211	2
15	32311	12311	10
16	32311	11211	2
17	21111	12211	50
18	32313	32331	50
19	11211	22211	4
20	23313	11133	50
21	11121	22112	10
22	12223	13332	10
23	11312	11211	2
24	11332	11312	4
25	11332	11211	2
26	21222	33321	2
27	22222	13311	50
28	11112	22112	4
29	33212	32223	4

## Footnotes

1 Other studies have explored the effect of different types of scales (e.g., VAS scales, rating scales) on preferences and attitudes other than WTP, for example Lee et al. [38], Aguinis et al. [1], Hui and Triandis [33].

2 Here, the minimum and maximum values obtained using PS are primarily relevant as intervals surrounding OE values.

3 The 29 scenarios were obtained by combining 42 different EQ-5D states and four probability levels (i.e., 2, 4, 10, and 50 %), representing a fair spread of QALY gains across the utility plane. The scenarios were previously used in deriving the British [35] and Dutch [37] EQ-5D tariffs and used by Gyrd-Hansen [28] in her study of WTP per QALY in Denmark.

4 A visual aid using dots in explaining the concept of risk was demonstrated to increase the validity of WTP responses by Corso et al. [17]. Our design of this graphical explanation was similar to that used in the recent EuroVaQ project, see http://research.ncl.ac.uk/eurovaq/EuroVaQ_Final_Publishable_Report_and_Appendices.pdf.

5 The difference in OE-WTP values between PS-5 and PS-25 was approx. a factor three, but the difference in WTP per QALY values was approx. a factor 1.5. This was due to the “mean of ratios” method employed in the calculation (i.e., the mean of ratios calculated for each row of data), suggesting that respondents estimated OE-WTP values that are non-proportional to the size of the expected QALY gain. Employing the “ratio of means” method, on the contrary, would lead to a proportional difference (i.e., factor three), but would not account for the distribution of individual values.

6 With respect to the other variables presented in Table 3, the independent variable LN(health gain) is significant and positive, pointing to the theoretical validity of our findings, although non-proportional in relation, signaling scope insensitivity [8]. LN(income) was also significant: on average, in case a household income was 10 % higher, the respondents were willing to pay 6.9 % (i.e., 1.10^0.697^) extra for the offered health gain.

7 For instance, if the “true” WTP value of a respondent is €50 for the offered health gain and the PS ranges from €0 to €500 with only 4 value points (e.g., 1, 100, 250, and 500) then the respondent needs to round her “true” value to the closest value point, either €1 or €100. Obviously, taking the either estimate as respondent’s WTP will lead to information loss and yield inaccurate WTP values (if inferred only from the PS, without OE-WTP).
